# Commentary: Global research on the crosstalk between intestinal microbiome and colorectal cancer: a visualization analysis

**DOI:** 10.3389/fcimb.2026.1779577

**Published:** 2026-02-26

**Authors:** Yuh-Shan Ho, Maria Christidis

**Affiliations:** 1 CT HO Trend, Taipei City, Taiwan; 2 Department of Neurobiology, Care Sciences and Society, Karolinska Institutet, Huddinge, Sweden; 3 Department of Nursing Science, Sophiahemmet University, Stockholm, Sweden

**Keywords:** bibliometrics, citation, methodological transparency, research rigor, search strategies

Bibliometric analyses are increasingly used to map global research trends and methodological developments. Yang et al. (2023) recently published a bibliometric analysis entitled “Global research on the crosstalk between intestinal microbiome and colorectal cancer: A visualization analysis” in *Frontiers in Cellular and Infection Microbiology*. In their study, the authors constructed the dataset using searches conducted within the title (TI), abstract (AB), and author keywords (AK) fields of the Science Citation Index Expanded. The aim of this commentary is to highlight methodological considerations related to bibliometric data retrieval strategies, with a particular focus on transparency, reproducibility, and appropriate methodological citation. This commentary does not aim to replicate or extend the bibliometric analysis by Yang et al. (2023), but rather uses their study as an illustrative example to discuss methodological choices in bibliographic data collection and filtering that precede large-scale statistical analysis.

Historically, bibliometric data retrieval from the Web of Science relied primarily on topic (TS) and title (TI) searches. However, extensive reliance on the TS field often led to the inclusion of records retrieved solely through *Keywords Plus*, which are algorithmically generated and may not accurately reflect the central focus of an article. This practice has been shown to introduce substantial noise into bibliometric datasets and to compromise analytical validity.

To address this methodological concern, Ho’s research group proposed the “front page” filtering concept in 2011, initially implemented using Microsoft Excel (Wang and Ho, 2011). This approach was subsequently examined, refined, and formally validated in an SCI-EXPANDED journal (Fu et al., 2012). The core principle of this methodology is to confine bibliometric analyses to publications in which the queried terms appear explicitly in the title, abstract, or author keywords, thereby improving dataset relevance, transparency, and reproducibility.

Empirical evidence has consistently demonstrated that the application of the “front page” filter produces substantial differences in retrieved datasets across medical and interdisciplinary fields. Reported deviations include 49% in studies of macrophage polarization (Ho, 2019a), 31% in ankylosing spondylitis research (Ho, 2021), 29% in nanoparticle neurotoxicity investigations (Ho, 2019b), 25% in analyses of artificial intelligence and machine learning applications in kidney care (Ho et al., 2024), and 24% in research on the mTOR signaling pathway in liver disease (Ho, 2021). These findings highlight the critical influence of retrieval strategy on bibliometric outcomes.

Following subsequent enhancements to the Web of Science platform, specifically the ability to independently search title (TI), abstract (AB), author keywords (AK), and Keywords Plus (KP), an updated TI-AB-AK “front page” configuration was introduced in 2021 (Usman and Ho, 2021). This revised framework has since been widely adopted and is increasingly regarded as a methodological benchmark in contemporary bibliometric research.

It is noteworthy that Yang et al. employed the “front page” approach in a later bibliometric study published in *Discover Oncology* (Yang et al., 2024). Nevertheless, the foundational publications that originally proposed and validated this methodology were not cited. Appropriate acknowledgment of methodological origins is a cornerstone of academic integrity, enabling readers to understand the rationale, evolution, and limitations of analytical techniques (Ho, 2014).

Failure to cite seminal methodological contributions may obscure intellectual provenance and impede transparency, reproducibility, and future methodological refinement. Moreover, the reuse of established analytical frameworks without explicit attribution may raise concerns regarding ethical and methodological transparency as well as appropriate acknowledgment of prior work (Moss and Hunter, 1994; Noè and Batten, 2006).

In summary, although the work of Yang et al. (2023) offers insights into global research trends on the interaction between the intestinal microbiome and colorectal cancer, closer attention to methodological citation practices is necessary. The “front page” methodology represents a well-documented and systematically validated approach, and its application should be accompanied by proper reference to its original sources. This commentary is intended to support methodological rigor in bibliometric research rather than to question the scientific relevance of the findings themselves.
